# Supplementary material for: Transforming sustainable plant proteins into high performance lubricating microgels
Source: Nat Commun. 2023 Aug 7;14:4743. doi: 10.1038/s41467-023-40414-7 (PMC10406910; doi:10.1038/s41467-023-40414-7)
Supplement: Supplementary file 1 — Supplementary Information [file 41467_2023_40414_MOESM1_ESM.pdf]

Supplementary Information

Transforming sustainable plant proteins into high performance lubricating microgels

*Ben Kew[1], Melvin Holmes[1]***, Evangelos Liamas[1,2], Rammile Ettelaie[1], Simon D. Connell[3]**, Daniele Dini[4], Anwesha Sarkar[1]**

[1] Food Colloids and Processing Group, School of Food Science and Nutrition, University of Leeds, Leeds, LS2 9JT, UK

[2] Unilever Research & Development Port Sunlight, Quarry Road East, Bebington, Merseyside, CH63 3JW, UK

[3] Molecular and Nanoscale Physics Group, School of Physics and Astronomy, University of Leeds, Leeds, LS2 9JT, UK

[4] Department of Mechanical Engineering, Imperial College London, London, SW7 2AZ UK

Corresponding authors:

* Prof. Anwesha Sarkar

E-mail address: A.Sarkar@leeds.ac.uk (A. Sarkar)

** Dr. Dr. Simon Connell

E-mail address: s.d.a.connell@leeds.ac.uk (S. Connell).

*** Dr. Mel Holmes

E-mail address: prcmjh@leeds.ac.uk (M. Holmes).

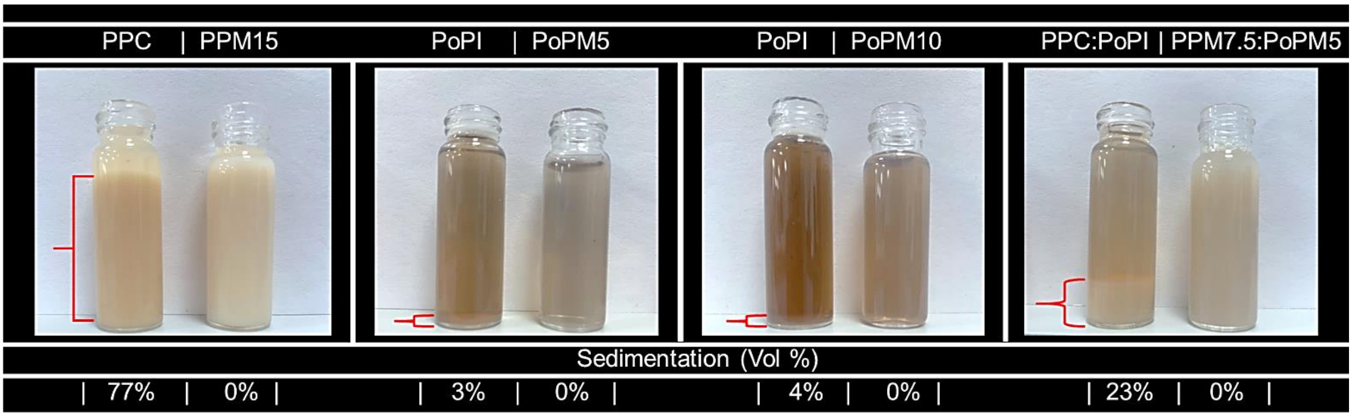

Supplementary Figure 1| Sedimentation in native plant protein solutions as compared to corresponding microgel dispersions. Images and sedimentation (vol %) of native plant protein and plant protein microgel solutions prepared using pea protein concentrate to form a 15.0 wt% total protein microgel, (PPM15), potato protein isolate to form a 5.0 wt% total protein microgel (PoPM5), potato protein isolate to form a 10.0 wt% total protein microgel, (PoPM10), and using a mixture of pea protein concentrate at 7.5 wt% total protein and potato protein isolate at 5.0 wt% total protein microgel (PPM7.5:PoPM5). Microgels were prepared with volume fraction Φ = 40 and compared to native protein solutions of the same protein concentration. Photographs were captured when native proteins were left for 1 day whilst microgels were left for 28 days, such stability in microgels persisted for months. Red brackets highlight sedimentation in native protein solutions compared to undetectable levels present in microgel solutions.

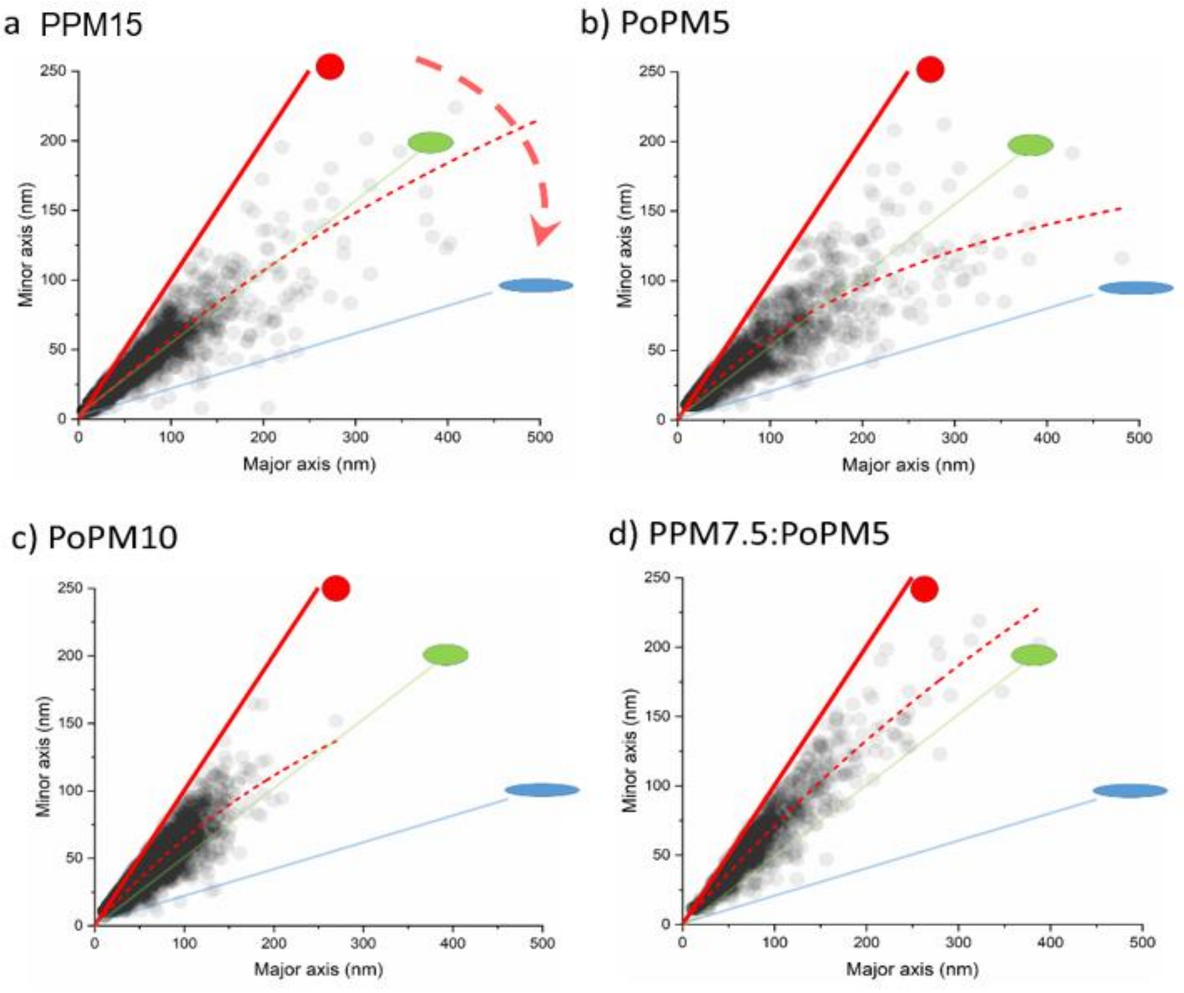

Supplementary Figure 2| Shape analysis of microgel particle size distribution. Shape analysis performed on several thousand microgels per sample shown in Fig 3, microgels prepared using (a) pea protein concentrate to form a 15.0 wt% total protein microgel, (PPM15), (b) potato protein isolate to form a 5.0 wt% total protein microgel (PoPM5), (c) potato protein isolate to form a 10.0 wt% total protein microgel, (PoPM10), and (d) a mixture of pea protein concentrate at 7.5 wt% total protein and potato protein isolate at 5.0 wt% total protein. Ellipses were fitted to each microgel, and the graph plots the short or minor axis vs the long or major axis. Hence, the perfectly spherical microgels will follow the red line with a 1:1 aspect ratio. Most microgels in all samples were between spherical and a 2:1 aspect ratio represented by the green line. A general trend found is the increase in aspect ratio as particle size increases, represented the dashed red fit lines (note: the Michaelis-Menten fit equation used is not physically relevant). This could be explained by the larger microgels being randomly shaped aggregates of the smaller microgels.

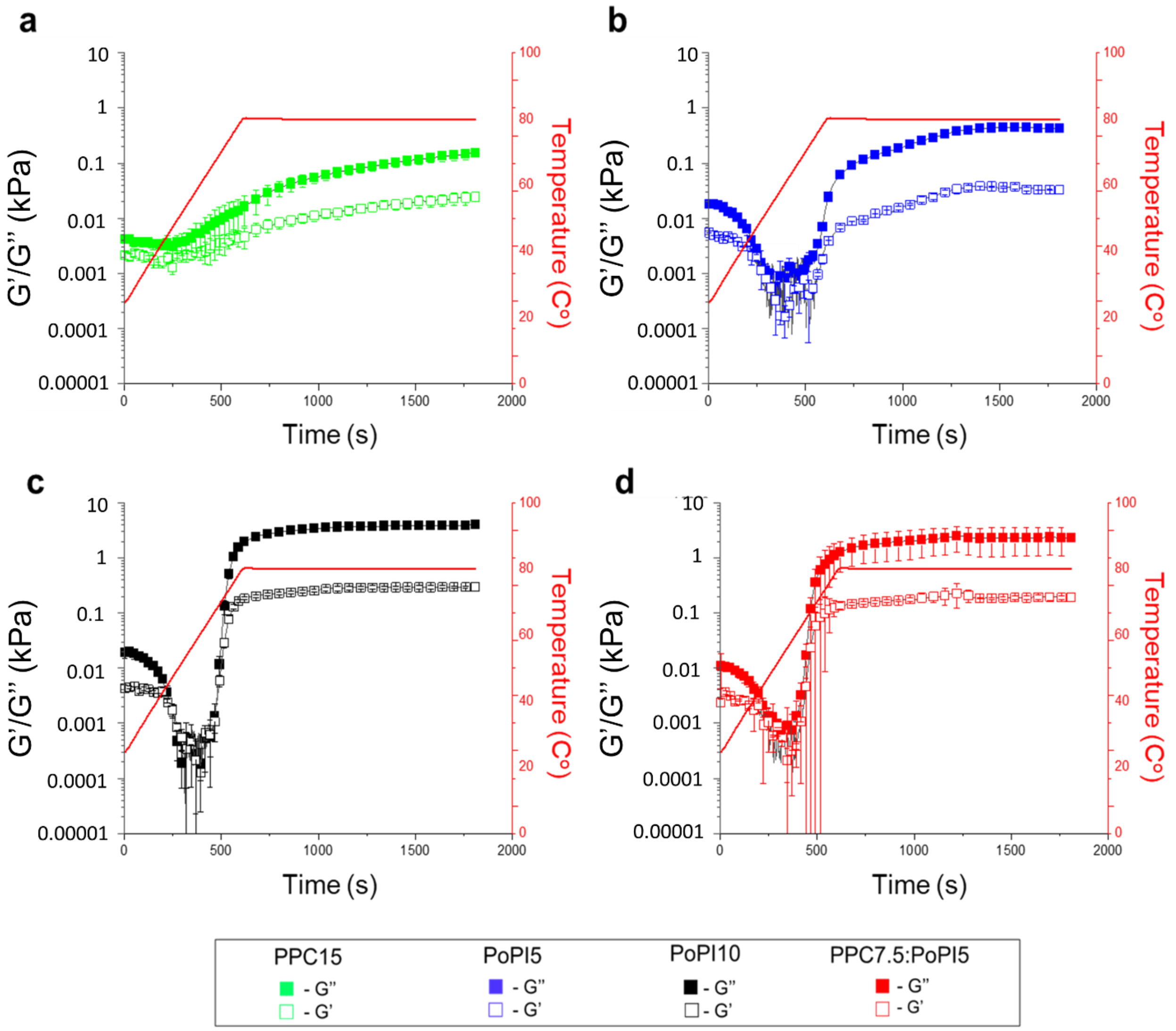

Supplementary Figure 3| Gelation properties of microgel parent protein gels. Temperature ramp with constant strain (0.1% at 1Hz) applied to native protein solutions prepared showing storage (G') and loss (G'') moduli of **(a)** pea protein concentrate to form a 15.0 wt% total protein microgel, (PPM15), **(b)** potato protein isolate to form a 5.0 wt% total protein microgel (PoPM5),**(c)** potato protein isolate to form a 10.0 wt% total protein microgel, (PoPM10), and **(d)** using a mixture of pea protein concentrate at 7.5 wt% total protein and potato protein isolate at 5.0 wt% total protein microgel (PPM7.5:PoPM5).Results are plotted as average of three repeat measurements on triplicate samples (n = 3 × 2) with error bars representing standard deviations.

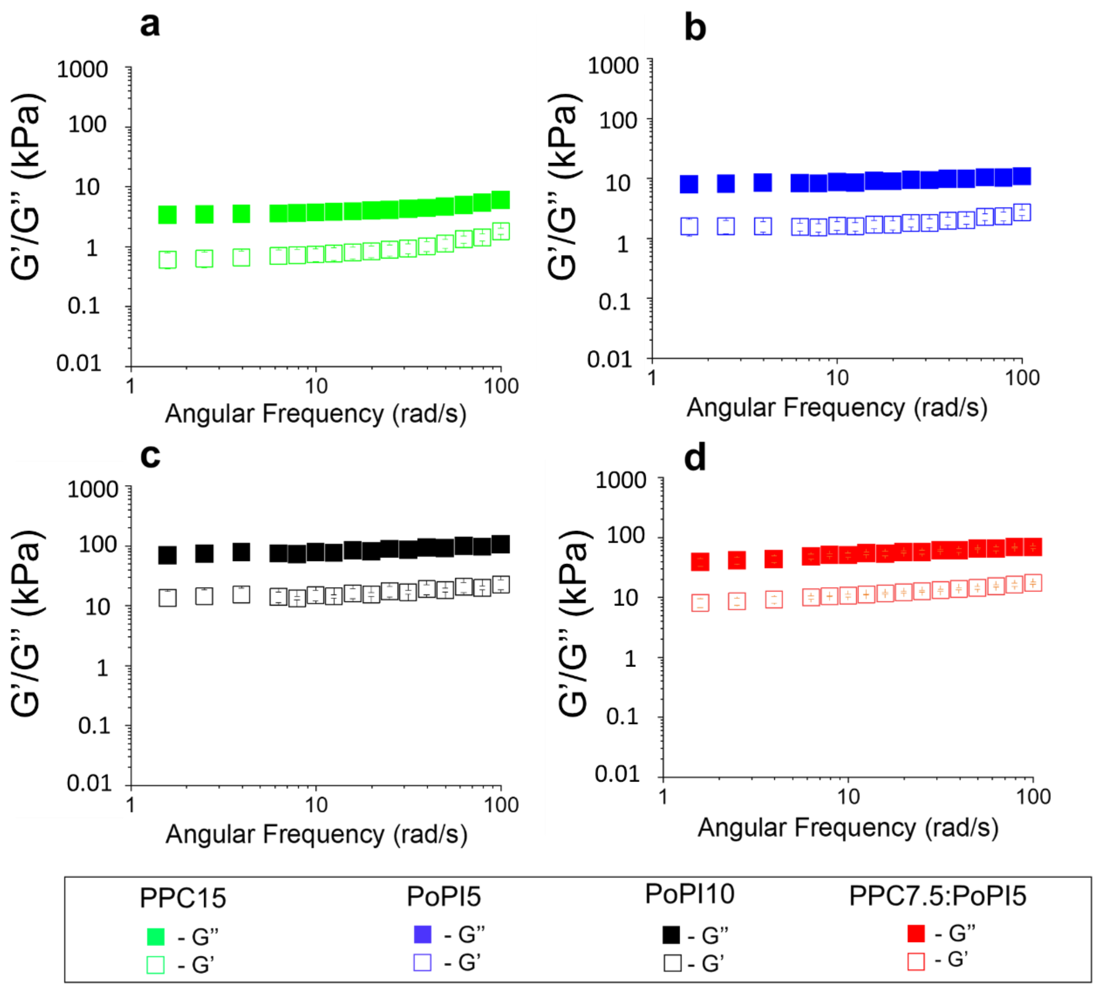

Supplementary Figure 4| Viscoelasticity of plant protein-based parent hydrogels. Frequency sweep of heat-set gels prepared using **(a)** pea protein concentrate to form a 15.0 wt% total protein microgel, (PPM15), (**b)** potato protein isolate to form a 5.0 wt% total protein microgel (PoPM5), (**c)** potato protein isolate to form a 10.0 wt% total protein microgel, (PoPM10), and **(d)** using a mixture of pea protein concentrate at 7.5 wt% total protein and potato protein isolate at 5.0 wt% total protein microgel (PPM7.5:PoPM5).Protein dispersions were added onto a cone-and-plate geometry (diameter 50 mm, angle 1°), and gelation was initiated using a temperature ramp (25 – 80°C at a rate of 0.08 ^{o}C/ s and held at 80 ^{o}C for 30 min) and cooled to 37 ^{o}C where a frequency sweep at a strain of 0.1% was initialised. Results are plotted as average of six measurements on triplicate samples (n = 6 × 3) with error bars representing standard deviations.

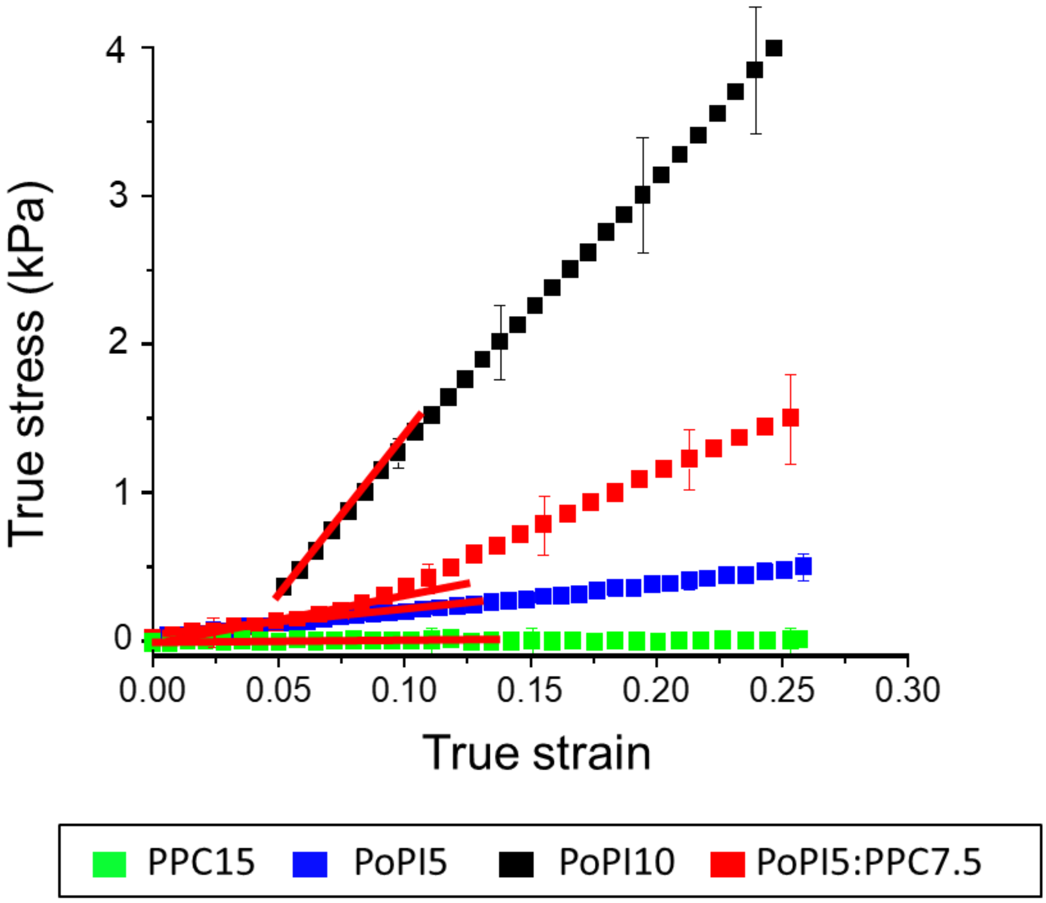

Supplementary Figure 5| Large scale deformation of the parent protein gels. Stress-strain curves of plant protein gels prepared using pea protein concentrate to form a 15.0 wt% gel, (PPM15), potato protein isolate to form a 5.0 wt% gel (PoPM5), potato protein isolate to form a 10.0 wt% gel, (PoPM10), and using a mixture of pea protein concentrate at 7.5 wt% total protein and potato protein isolate at 5.0 wt% total protein gel (PPM7.5:PoPM5). Values where Young's modulus was calculated is indicated by the red vector. Results are plotted as average of three repeat measurements on triplicate samples (n = 3 × 2) with error bars representing standard deviations.

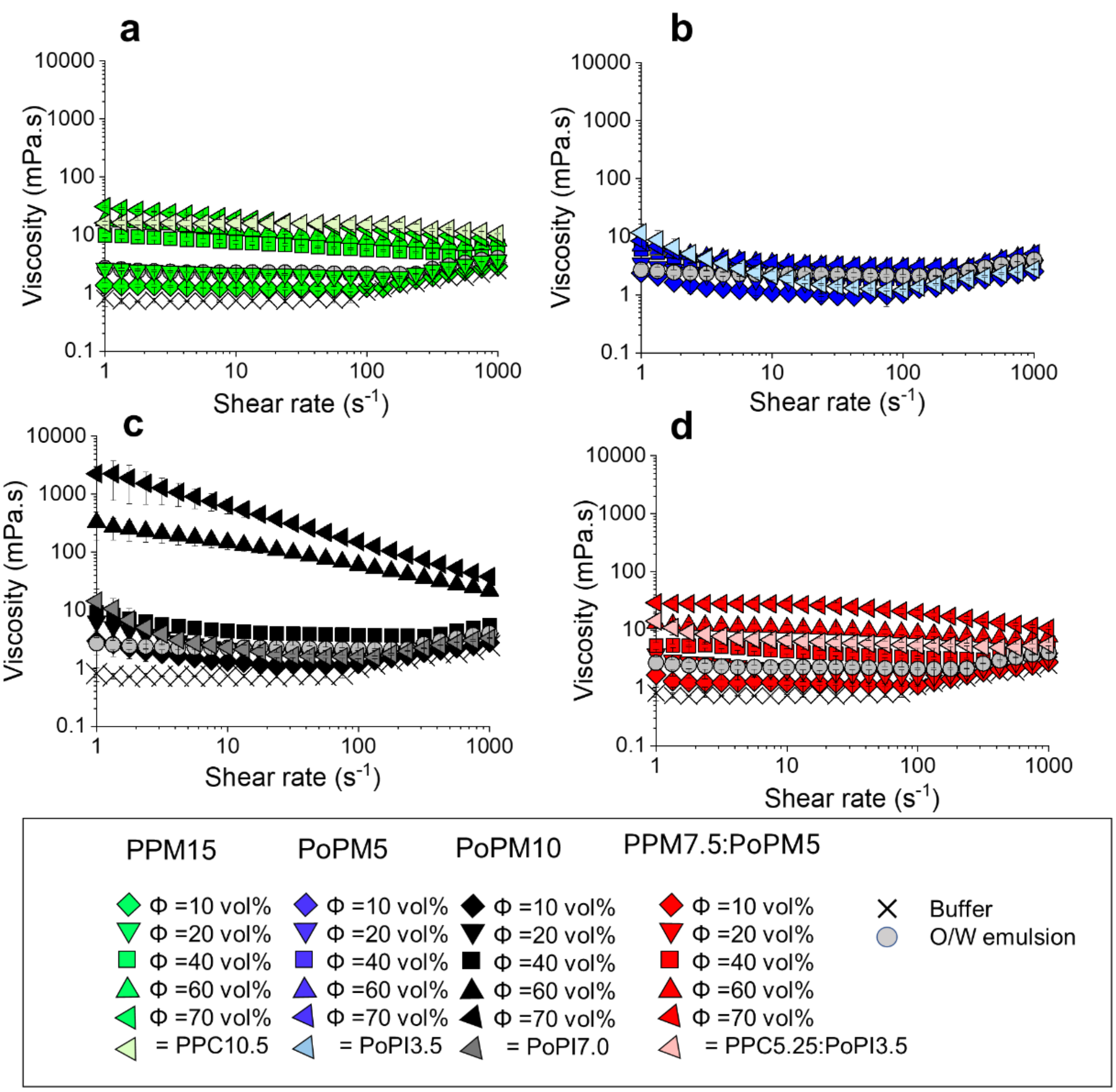

Supplementary Figure 6| Flow curves of plant protein microgels. Apparent viscosity of plant protein microgels prepared using (**a**) pea protein concentrate to form a 15.0 wt% total protein microgel, (PPM15) (**b**) potato protein isolate to form a 5.0 wt% total protein microgel (PoPM5), (**c**) potato protein isolate to form a 10.0 wt% total protein microgel, (PoPM10) and (**d**) using a mixture of pea protein concentrate at 7.5 wt% total protein and potato protein isolate at 5.0 wt% total protein microgel (PPM7.5:PoPM5), as function of shear rate, respectively. Plant protein microgels were compared to native proteins (matched protein content for Φ =70 vol% with numbers displayed relating to the total protein content). 20.0 wt% oil-in-water (O/W) emulsion and buffer are shown in each plots (**a-d**) as controls. Shear rates were measured from ramping up from 1 s^{-1} to 1000 s^{-1} at 37 °C with plots as average of six measurements on triplicate samples (n = 6 × 3) with error bars representing standard deviations.

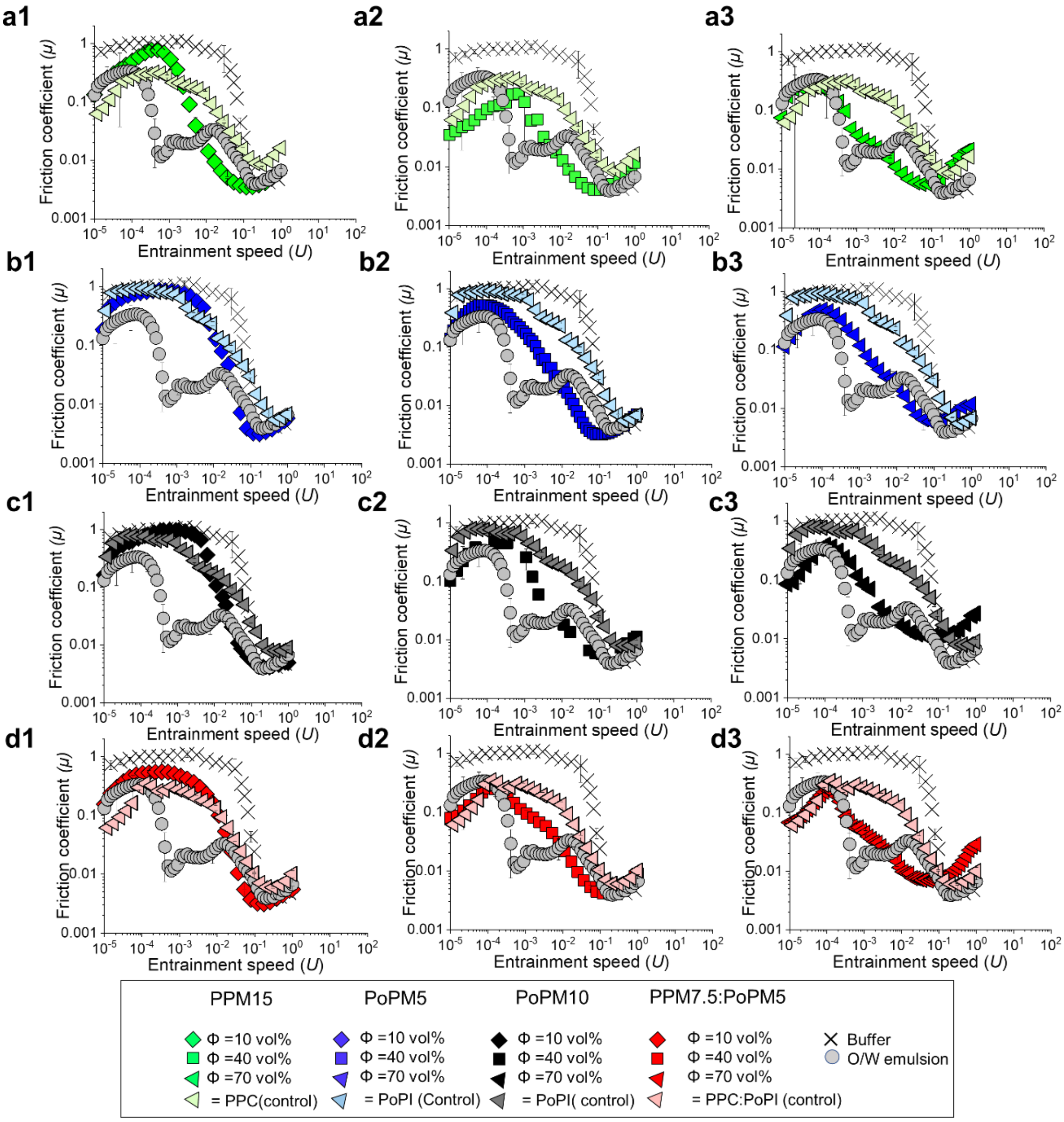

Supplementary Figure 7| Frictional behaviour in hard-soft contact surfaces in presence of plant protein microgels. Tribological performance of steel ball on PDMS surfaces in the presence of plant protein microgels, native plant protein (matched protein content for Φ =70 vol% with numbers displayed relating to total protein content or oil-in-water emulsion. Friction coefficient (μ) as a function of entrainment in the presence of plant protein microgels prepared using (**a1-3**) pea protein concentrate to form a 15.0 wt% total protein microgel, (PPM15), (**b1-3**) potato protein isolate to form a 5.0 wt% total protein microgel (PoPM5), (**c1-3**)), potato protein isolate to form a 10.0 wt% total protein microgel, (PoPM10), and (**d1-3**) using a mixture of pea protein concentrate at 7.5 wt% total protein and potato protein isolate at 5.0 wt% total protein microgel (PPM7.5:PoPM5) with 1, 2 and 3 showing increased volume fractions from 10 to 70 vol%, respectively. Frictional responses of the plant proteins at the highest concentration and 20 wt% oil-in-water emulsion (O/W emulsion) and buffer are included in each graph (**a-d**) as controls. Results are plotted as average of six repeat measurements on triplicate samples (n = 6 × 3) with error bars representing standard deviations.

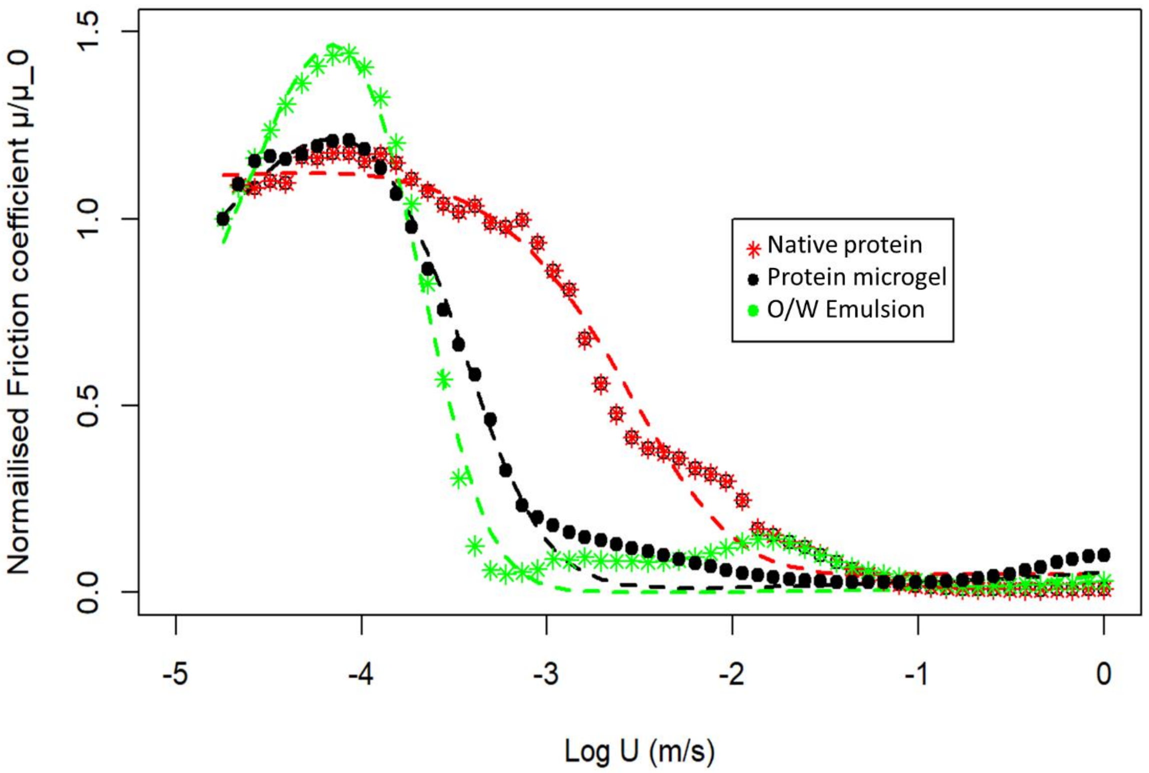

Supplementary Figure 8| Normalised friction force of microgels compared against emulsions and native protein. Tribological performance of hard/soft (steel ball-on-PDMS) contact surfaces showing theoretical modelling of the normalised ratio of friction coefficients against the initial level at a load of 2.0 N. Microgel and emulsions show close resemblance in friction coefficients in comparison to those obtained in the presence of native protein, in the latter a greater speed is required before a reduction in normalized friction is observed. Here the dashed lines show the best theoretical fit using **equation 3** in the main text.

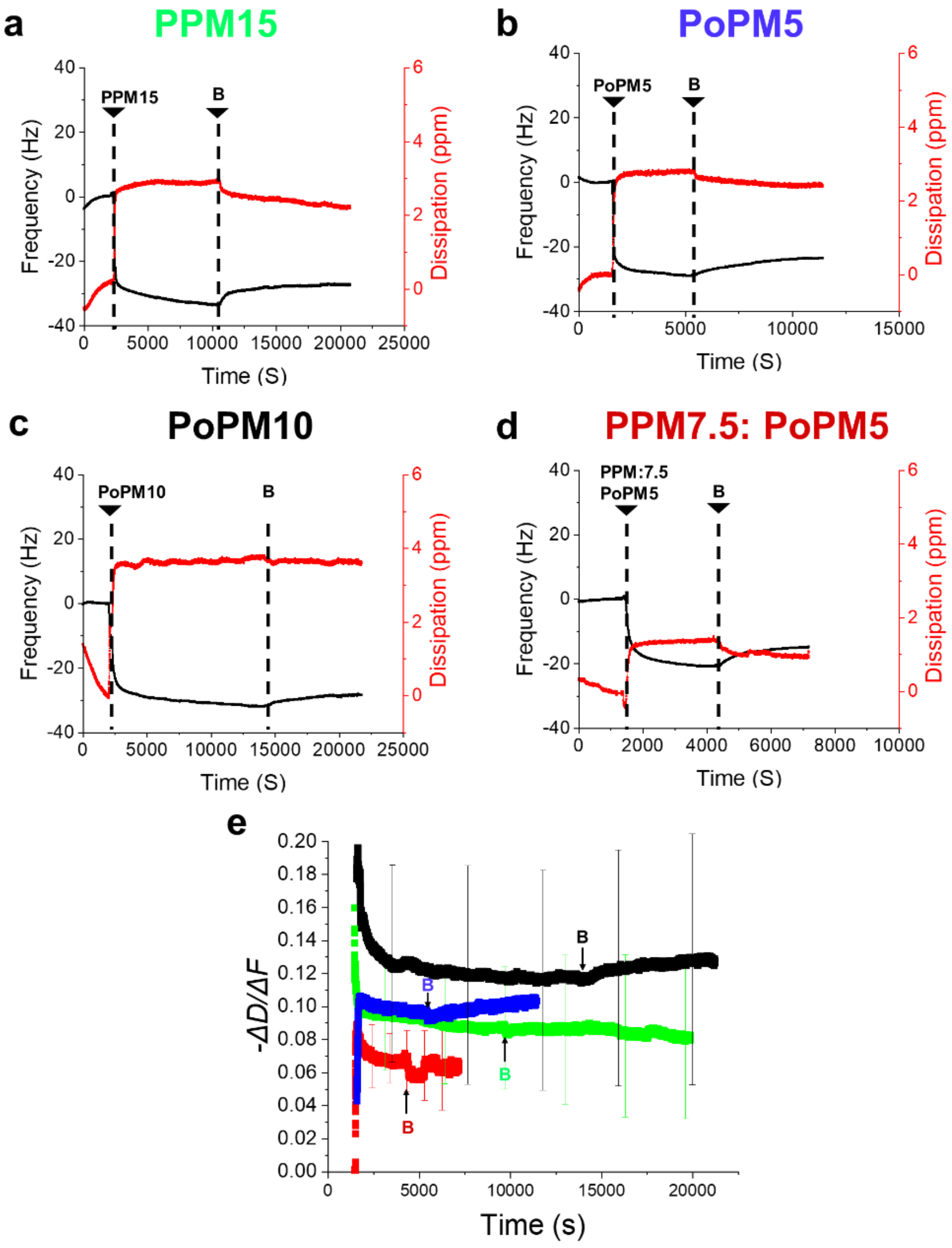

Supplementary Figure 9| Adsorption properties of plant protein microgels. Mean frequency and dissipation (5th overtone shown) of plant protein microgels prepared using **(a)** pea protein concentrate to form a 15.0 wt% total protein microgel, (PPM15), **(b)** potato protein isolate to form a 5.0 wt% total protein microgel (PoPM5), **(c)** potato protein isolate to form a 10.0 wt% total protein microgel, (PoPM10), and **(d)** using a mixture of pea protein concentrate at 7.5 wt% total protein and potato protein isolate at 5.0 wt% total protein microgel (PPM7.5:PoPM5) on PDMS-coated hydrophobic sensors with B implying injection of HEPES buffer. Viscoelasticity of the films (**e**) is represented by dissipation shift (ΔD)/frequency shift ratio (Δ*f*) i.e. –ΔD/Δ*f* of protein microgel solutions with step B representing the final buffer rinse stage. Results are plotted as average of three repeat measurements on triplicate samples (n = 3 × 2) with error bars representing standard deviations.

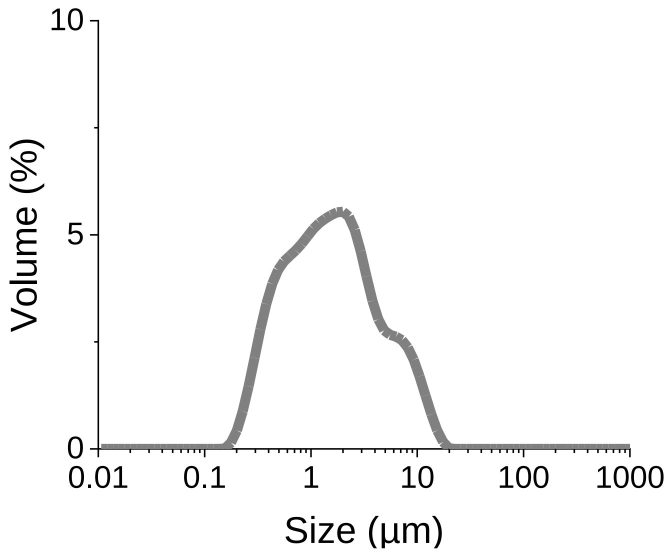

Supplementary Figure 10| Droplet size distribution of 20:80 O/W Emulsion. Mean droplet size distribution of 20:80 O/W emulsions stabilised using 1.5 wt% potato protein. Results are plotted as average of three repeat measurements on triplicate samples (n = 3 × 2).

Supplementary Table 1| Evolution of size of plant protein microgels with time. Mean and standard deviation (SD) of the change in hydrodynamic diameter (d_H) of plant protein microgels as a function of storage time (T, days) at room temperature (22 ºC). Plant protein microgels were prepared using pea protein concentrate to form a 15.0 wt% total protein microgel, (PPM15), potato protein isolate to form a 5.0 wt% total protein microgel (PoPM5), potato protein isolate to form a 10.0 wt% total protein microgel, (PoPM10), and using a mixture of pea protein concentrate at 7.5 wt% total protein and potato protein isolate at 5.0 wt% total protein microgel (PPM7.5:PoPM5). Different lower-case letters in the same column indicate a statistically significant difference ($p < 0.05$).

	**PPM15**		**PoPM5**		**PoPM10**		**PPM7.5:PoPM5.0**	
**Storage time (Days)**	d_H	**PDI**	d_H	**PDI**	d_H	**PDI**	d_H **(Peak 1)**	d_H **(Peak 2)**
**T = 0**	204 ± 7 ^{a}	0.22 ± 0.02 ^{a}	60 ± 1 ^{a}	0.23 ± 0.01 ^{a}	132 ± 2.5 ^{a}	0.21 ± 0.01 ^{a}	238 ± 4 ^{a}	48 ± 12 ^{a}
**T = 7**	236 ± 5 ^{a}	0.30 ± 0.04 ^{s}	67 ± 5 ^{ab}	0.27 ± 0.01 ^{b}	89.7 ± 4 ^{b}	0.22 ± 0.01 ^{ab}	260 ± 14 ^{b}	100 ± 10 ^{b}
**T = 14**	210 ± 5 ^{a}	0.24 ± 0.03 ^{a}	72 ± 2 ^{b}	0.25 ± 0.01 ^{b}	98 ± 2 ^{c}	0.24 ± 0.01 ^{b}	300 ± 10 ^{c}	96 ± 13 ^{b}
**T = 28**	222 ± 6 ^{a}	0.26 ± 0.02 ^{a}	78 ± 1 ^{b}	0.24 ± 0.01 ^{b}	105 ± 7 ^{a}	0.25 ± 0.01 ^{b}	315 ± 15 ^{c}	115 ± 17 ^{b}

Supplementary Table 2| Stability of plant protein microgels when subjected to processing. Mean and standard deviation (SD) of the change in hydrodynamic diameter of of plant protein microgels when heated at 90 ^{o}C for 30 min. Plant protein microgels were prepared using pea protein concentrate containing 15.0 wt% protein (PPM15), potato protein isolate containing 5.0 wt% protein (PoPM5), potato protein isolate containing 10.0 wt% protein (PoPM10), and mixed pea protein concentrate containing 7.5 wt% protein and potato protein isolate containing 5.0 wt% protein (PPM7.5:PoPM5). Different lower-case letters in the same column indicate a statistically significant difference ($p < 0.05$).

	**PPM15**		**PoPM5**		**PoPM10**		**PPM7.5:PoPM5.0**	
**Time (s)**	d_H	PDI	d_H	PDI	d_H	PDI	d_H (Peak 1)	d_H (Peak 2)
**T = 0**	204 +/- 7 ^{a}	0.22 +/- 0.01 ^{a}	60 +/- 1 ^{a}	0.23 +/- 0.01 ^{a}	115 +/- 5	0.21 +/- 0.01 ^{a}	238 +/- 4 ^{a}	48 +/- 12 ^{a}
**T = 600**	211 +/- 5 ^{a}	0.25 +/- 0.01 ^{b}	76 +/- 1 ^{b}	0.23 +/- 0.01 ^{a}	119 +/- 1	0.24 +/- 0.01 ^{b}	239 +/- 8 ^{a}	48 +/- 9 ^{a}
**T = 1800**	198 +/- 3 ^{a}	0.22 +/- 0.02 ^{a}	86 +/- 2 ^{c}	0.24 +/- 0.01 ^{a}	130 +/- 5	0.23 +/- 0.01 ^{b}	235 +/- 7 ^{a}	56 +/- 5 ^{a}

Supplementary Table 3| Comparison of frictional coefficients of plant protein microgels in hard-soft contact surfaces. Mean and standard deviation (SD) of the friction coefficients of plant protein microgels prepared using **(a)** pea protein concentrate to form a 15.0 wt% total protein microgel, (PPM15), **(b)** potato protein isolate to form a 5.0 wt% total protein microgel (PoPM5), **(c)** potato protein isolate to form a 10.0 wt% total protein microgel, (PoPM10), and **(d)** using a mixture of pea protein concentrate at 7.5 wt% total protein and potato protein isolate at 5.0 wt% total protein microgel (PPM7.5:PoPM5). Different Φ of microgels were compared against buffer, native protein (at Φ = 70 equivalent) and O/W emulsion. Different lower-case letters in the same column indicate a statistically significant difference ($p < 0.05$).

**Coefficient of friction of PPM15**		
	Mixed lubrication regime (0.1 Pa m)	
	Mean	SD
Φ =10 vol%	0.00573^{a}	0.001
Φ =40 vol%	0.00893^{b}	0.00122
Φ =70 vol%	0.01096^{c}	0.001
Buffer	0.744^{d}	0.300
O/W emulsion	0.02771^{e}	0.00533
PPC10.5	0.1358^{f}	0.01681
**Coefficient of friction of PoPM5**		
	Mixed lubrication regime (0.1 Pa m)	
	Mean	SD
Φ =10 vol%	0.01193^{a}	0.0027
Φ =40 vol%	0.0064^{b}	0.00269
Φ =70 vol%	0.01296^{a}	0.00388
Buffer	0.744^{c}	0.300
O/W emulsion	0.02771^{d}	0.00533
PoPI3.5	0.06511^{e}	0.01022
**Coefficient of friction of PoPM10**		
	Mixed lubrication regime (0.1 Pa m)	
	Mean	SD
Φ =10 vol%	0.01084^{a}	0.00119
Φ =40 vol%	0.0116^{a}	0.00166
Φ =70 vol%	0.03457^{b}	0.00597
Buffer	0.744^{c}	0.300
O/W emulsion	0.02771^{d}	0.00533
PoPI7.0	0.08773^{e}	0.00882
**Coefficient of friction of PPM7.5:PoPM5**		
	Mixed lubrication regime (0.1 Pa m)	
	Mean	SD
Φ =10 vol%	0.01063^{a}	0.001
Φ =40 vol%	0.01177^{b}	0.001
Φ =70 vol%	0.01161^{b}	0.0028
Buffer	0.744^{c}	0.300
O/W emulsion	0.02771^{d}	0.00533
PPC5.25: PoPI3.5	0.06172^{e}	0.00192

Supplementary Table 4| Comparison of frictional coefficients of plant protein microgels at a range of speeds in 3D biomimetic tongue-like surfaces. Mean and standard deviation (SD) of the friction coefficients of plant protein microgels prepared using **(a)** pea protein concentrate to form a 15.0 wt% total protein microgel, (PPM15), **(b)** potato protein isolate to form a 5.0 wt% total protein microgel (PoPM5), **(c)** potato protein isolate to form a 10.0 wt% total protein microgel, (PoPM10), and **(d)** using a mixture of pea protein concentrate at 7.5 wt% total protein and potato protein isolate at 5.0 wt% total protein microgel (PPM7.5:PoPM5) in the boundary and mixed regimes. Different Φ of microgels were compared against buffer and O/W emulsion. Different lower-case letters in the same row indicate a statistically significant difference ($p < 0.05$).

	**Buffer**		**20% O/W emulsion**		**PPM15**						**PoPM5**					
**VR (M/S)**		**SD**		**SD**	**10**	**SD**	**40**	**SD**	**70**	**SD**	**10**	**SD**	**40**	**SD**	**70**	**SD**
0.0005	0.56^{a}	0.11	0.30^{b}	0.08	0.26^{cb}	0.04	0.20^{d}	0.03	0.20^{d}	0.02	0.50^{a}	0.08	0.48^{a}	0.03	0.31^{e}	0.04
0.001	0.65^{a}	0.13	0.24^{b}	0.03	0.28^{cb}	0.03	0.25^{cb}	0.03	0.24^{c}	0.03	0.55^{a}	0.05	0.41^{d}	0.01	0.34^{e}	0.05
0.002	0.64^{a}	0.10	0.30^{b}	0.03	0.30^{cb}	0.02	0.27^{cb}	0.01	0.23^{d}	0.01	0.60^{a}	0.11	0.55^{ab}	0.06	0.36^{e}	0.04
0.0050	0.70^{a}	0.14	0.36^{b}	0.03	0.38^{cb}	0.04	0.31^{d}	0.01	0.28^{e}	0.02	0.59^{f}	0.05	0.45^{c}	0.04	0.40^{bc}	0.06
0.008	0.70^{a}	0.13	0.39^{b}	0.04	0.43^{cb}	0.04	0.36^{db}	0.02	0.33^{d}	0.03	0.62^{a}	0.07	0.46^{c}	0.05	$0.40^{c,b}$	0.05
0.0099	0.64^{a}	0.07	0.39^{b}	0.01	0.44^{b}	0.05	0.38^{cb}	0.02	0.36^{c}	0.02	0.67^{a}	0.10	0.56^{a}	0.09	$0.41^{b,c}$	0.05
0.02	0.69^{a}	0.08	0.39^{b}	0.02	0.45^{c}	0.03	0.42^{b}	0.01	0.39^{b}	0.04	0.45^{c}	0.05	0.41^{b}	0.05	0.41^{b}	0.04
0.0495	0.81^{a}	0.12	0.39^{b}	0.02	0.40^{b}	0.03	0.44^{b}	0.03	0.42^{b}	0.03	0.49^{c}	0.02	0.44^{b}	0.04	0.41^{b}	0.03
0.0798	0.77^{a}	0.12	0.40^{b}	0.02	0.36^{b}	0.02	0.47^{c}	0.02	0.45^{c}	0.05	0.44^{c}	0.04	0.35^{d}	0.01	0.42^{b}	0.03

	**PoPM10**						**PoPM5:PPM7.5**					
**VR (M/S)**	**10**	**SD**	**40**	**SD**	**70**	**SD**	**10**	**SD**	**40**	**SD**	**70**	**SD**
0.0005	0.43^{a}	0.06	0.38^{e}	0.05	0.22^{cd}	0.03	0.21^{d}	0.02	0.37^{e}	0.06	0.40^{abce}	0.19
0.001	0.48^{d}	0.06	0.42^{d}	0.05	$0.26^{b,c}$	0.05	$0.27^{b,c}$	0.04	0.41^{d}	0.08	0.47^{d}	0.12
0.002	0.50^{a}	0.09	0.45^{e}	0.06	0.30^{cf}	0.06	0.30^{cf}	0.02	0.46^{be}	0.12	0.55^{ae}	0.17
0.00501	0.55^{f}	0.06	0.50^{c}	0.04	0.38	0.10	0.34^{cd}	0.04	0.45^{bc}	0.10	0.65^{a}	0.09
0.008	0.59^{a}	0.08	0.52^{c}	0.05	0.43^{bcd}	0.15	0.34^{d}	0.02	0.46^{c}	0.09	0.68^{a}	0.12
0.0099	0.53^{b}	0.09	0.49^{b}	0.03	0.48^{bcde}	0.21	0.35^{d}	0.04	0.48^{bde}	0.09	0.55^{e}	0.03
0.02	0.53^{c}	0.05	0.40^{b}	0.02	0.53^{abc}	0.21	0.35^{d}	0.01	0.46^{bc}	0.08	0.61^{a}	0.05
0.0495	0.53^{c}	0.10	0.41^{b}	0.01	0.64^{c}	0.16	0.34^{d}	0.01	0.41^{b}	0.07	0.61^{e}	0.01
0.0798	0.49^{c}	0.07	0.40^{b}	0.01	0.68^{a}	0.19	0.32^{d}	0.02	0.39^{bc}	0.14	0.69^{a}	0.07

Supplementary information 1| Theoretical analysis of indentation and drag force of lubricants.

The total load (W_T) in mixed regime is supported by both lubricant and the asperities. As lubricant was substantially lower in friction than the buffer, the following expression was used to calculate load:

$$W_L = \frac{\mu B - \mu}{\mu B} W_T \quad (1)$$

where, μ is the friction of buffer or lubricant at a defined entrainment speed.

To understand the physical properties of lubricant separating contact surfaces, the mechanical analysis can be performed using Hertz theory at the contact point. The radius (a_H) and the indentation δ are obtained using equations 2 and 3 for a point of contact supporting load W.

$$a_H{}^3 = \frac{3}{4}\frac{WR^*}{E^*} \quad (2)$$

$$\delta = \frac{a_H{}^2}{R^*} - f\left(\frac{a_H}{R}\right)\frac{W}{\pi R^* E(1-v^2)} \quad (3)$$

With $f\left(\frac{a_H}{R}\right) = \frac{2\,(1+v)}{\left(4+\left(\frac{a_H}{R}\right)^2\right)^{3/2}} + \frac{(1-v^2)}{\left(4+\left(\frac{a_H}{R}\right)^2\right)^{1/2}}$ (4)

R* represents reduced radius $\left(\frac{1}{R\prime}+\frac{1}{R\prime\prime}\right)^{-1}$ from PDMS and steel ball contact, v is the Poisson ratio and E^* the elastic modulus between lubricant and PDMS surface. E^*, obtained from the expression $\left(\frac{1-v^2}{E\prime}+\frac{1-v^2}{E\prime\prime}\right)^{-1}$ is the reduced elastic modulus of PDMS and lubricant. For the microgels the elastic modulus measured from heated parent plant proteins was used and for emulsion this was estimated from $E = 2G'_f(1+v)$ where $G'_f = \frac{2y}{R}$ where y is the interfacial surface tension of oil stabilised by potato protein measured at y = 27 mN m^{-1} with R being radius of particle.

The load supported by each lubricant (W_p) was estimated using the following equation where $a_{tp}{}^2$ is the Hertz contact radius between glass ball and PDMS calculated using equation 2 :

$$Wp = \frac{W_p}{N_p} = \frac{1}{\emptyset_p}\frac{W_T R^2}{a_{tp}{}^2} \quad (5)$$

Combining equations 5, 2 and 3, the relative indentation of the monolayer of microgel particles or emulsion can be expressed as:

$$\frac{\delta}{R} = \left(\frac{a_H}{R}\right)^2 - \frac{4}{3\pi(1-v^2)}\left(\frac{a_H}{R}\right)^3 f\left(\frac{a_H}{R}\right) \quad (6)$$

Where, a_H/R is independent of R and is the relative indentation of the surface particles independent of particle radius

$$\frac{a_H}{R} = \left(\frac{3W_L}{4\emptyset_p E^* atp^2}\right)^{1/3} \quad (7)$$
